# Palmitate Promotes the Paracrine Effects of Macrophages on Vascular Smooth Muscle Cells: The Role of Bone Morphogenetic Proteins

**DOI:** 10.1371/journal.pone.0029100

**Published:** 2012-02-21

**Authors:** Ji Hyung Chung, Hyun Ju Jeon, Sung-Yu Hong, Da Lyung Lee, Kyung Hye Lee, Soo Hyuk Kim, Ye Sun Han, Ichiro Manabe, Yury I. Miller, Sang-Hak Lee

**Affiliations:** 1 Cardiovascular Product Evaluation Center, Cardiovascular Research Institute, Yonsei University Health System, Seoul, Korea; 2 Interdisciplinary Course of Science for Aging, Graduate School, Yonsei University, Seoul, Korea; 3 National Research Laboratory for Cardiovascular Therapy, Biobud Inc., Seoul, Korea; 4 Yonsei Research Institute of Aging Science, Yonsei University, Seoul, Korea; 5 Department of Advanced Technology Fusion, Konkuk University, Seoul, Korea; 6 Department of Cardiovascular Medicine, Graduate School of Medicine, The University of Tokyo, Tokyo, Japan; 7 Department of Medicine, University of California San Diego, La Jolla, California, United States of America; 8 Cardiology Division, Department of Internal Medicine, Yonsei University College of Medicine, Seoul, Korea; Universität Würzburg, Germany

## Abstract

Saturated fatty acids are known to activate macrophages and induce vascular inflammation. Although cytokines from activated macrophage influence other vascular cells, the influence of saturated fatty acids on the paracrine effect of macrophages is not fully understood yet. Here we examined the impact of palmitate on the effect of macrophages on vascular smooth muscle cells (SMCs) and their mediators. SMCs proliferation increased significantly after treatment with conditioned media from palmitate-stimulated RAW264.7 cells. SMC migration was found to be greater after treatment with palmitate-conditioned media. SM α-actin and SM22α were decreased in SMCs treated with palmitate-conditioned media. When stimulated with palmitate, RAW264.7 cells secreted more bone morphogenetic protein (BMP)2 and BMP4 into the cell culture media. SMC proliferation, migration, and phenotypic changes were attenuated after treatment of neutralizing antibodies against BMPs or knockdown of BMPs with siRNA. The influences of these proteins were further confirmed by direct treatment of recombinant BMP2 and BMP4 on SMCs. Particularly, the effects of BMPs on SMC migration on phenotypic change were obvious, whereas their effect on SMC proliferation seemed not significant or modest. In conclusion, palmitate promoted macrophages' paracrine effects on SMC proliferation, migration, and phenotypic change. The effect of stimulated macrophages was mediated, at least in part, by BMP2 and BMP4. These results suggest a novel mechanism linking saturated fatty acids and the progression of vascular diseases that is possibly mediated by BMPs from macrophages.

## Introduction

Free fatty acid levels are often elevated in obese individuals and patients with metabolic syndrome or diabetes, and predicts cardiovascular events [1]. Although the mechanisms by which free fatty acids affect vascular diseases such as atherosclerosis are not completely understood, a growing body of evidence suggests that they are involved in the promotion of vascular inflammation. In particular, saturated fatty acids have been reported to activate monocytes/macrophages and induce the production of several inflammatory mediators such as tumor necrosis factor- α (TNF- α), interleukin-6 (IL-6), and interleukin-1β (IL-1β) [2]–[4].

The proliferation and phenotypic changes of vascular smooth muscle cells (SMCs) from a quiescent and contractile to a synthetic form are critical in atherosclerosis. SMCs interact with other vascular cells including endothelial cells, monocytes, and macrophages and these interactions can influence SMC phenotypes. Known factors involved in the modulation of SMC phenotypes include growth factors such as platelet-derived growth factor (PDGF), angiotensin II, interleukins, and mechanical stimulation [5].

Bone morphogenetic proteins (BMPs) constitute a large group in the transforming growth factor-β superfamily [6]. They are known for their key roles in signaling during embryogenesis and remodeling of bone and other tissues. BMP expression is upregulated in human atherosclerotic lesions [7] and is involved in vascular calcification and inflammation [8]. BMP4 is proinflammatory when expressed in endothelial cells [9]. Although the effects of BMP2 and BMP4 on vascular SMCs have been evaluated in a few studies of pulmonary vasculature, their relationships with SMCs are not fully understood [10]–[12].

Here, we examined the impact of palmitate on the paracrine effects of macrophages on vascular SMCs. We investigated the effects of palmitate-stimulated macrophages on SMC proliferation, migration, and phenotypic change. We hypothesized that BMPs could mediate macrophage-dependent SMC changes and demonstrated the role of BMP2 and 4 in the interactions between these two cell types.

## Methods

### Materials

Sodium salt of palmitate, bovine serum albumin (BSA; fatty acid-free and low endotoxin), phorbol 12-myristate 13-acetate (PMA), β-mercaptoethanol, mouse monoclonal antibodies against smooth muscle α-actin (SM α-actin) and SM22α were purchased from Sigma-Aldrich (St. Louis, MO, USA). Dulbecco's modified Eagle's medum (DMEM), RPMI 1640 medium, gentamycin, fetal bovine serum (FBS) and Dulbecco' phosphate buffered saline (PBS) with Ca^2+^ and Mg^2+^ were obtained from Gibco (Grand Island, NY, USA). Neutralizing antibodies against BMP2 and BMP4 were obtained from LSBio (Seattle, WA, USA) and Abcam (Cambridge, MA, USA), respectively. Isotype-matched control IgG and recombinant BMPs were purchased from R&D Systems (Minneapolis, MN, USA).

### Preparation of palmitate

Palmitate was dissolved in 0.1 M NaOH/70% ethanol at 70°C. It was then complexed with 10% fatty acid-free low endotoxin BSA at 55°C for 10 minutes. A stock solution of 50 mM palmitate was prepared before the experiment. Palmitate was used at a concentration of 250 µM and the solution was adjusted to a pH of 7.4. Palmitate preparation was assessed for lipopolysaccharide contamination with Limulus Amebocyte Lysate Assay (Lonza, Basel, Switzerland), and the endotoxin level was <0.05 EU/mL through all experiments. Control solution containing ethanol and BSA was prepared similarly.

### Cell culture

Rat aortic smooth muscle cells (SMCs) were obtained from BioBud Inc. (Seoul, Korea) and grown in DMEM containing 10% heat-inactivated FBS, 100 U/mL penicillin and 100 µg/mL streptomycin. RAW264.7 cells and human monocyte leukemia cell line THP-1 were purchased from the Korean Cell Line Bank (Seoul, Korea). RAW264.7 cells were grown in DMEM with 10% FBS. THP-1 cells were maintained in medium supplemented with 10% FBS and 0.2% β-mercaptoethanol.

### Bromodeoxyuridine (BrdU) incorporation assay

SMC proliferation was examined using a BrdU cell proliferation assay kit (Millipore Chemicon, Billerica, MA, USA) according to the manufacturer's protocol.RAW264.7 cells were stimulated with BSA control or palmitate (250 µM)for 4 hours. Conditioned media were collected 20 hours later. PDGF that is known to induce SMC proliferation [13] and phenotypic modulation [14] was used with IL-1β for a positive control group. SMCs plated in 96-well plates (3,000 cells/well) were serum-starved for 24 hours and treated with each conditioned media or PDGF (25 ng/mL)/IL-1β (10 ng/mL) for 24 to 72 hours. The cells labeled with BrdU for 4 hours and then fixed with fixing solution. The plates were washed for three times and stained with anti-BrdU antibody for 1 hour. After three washes, peroxidase-conjugated secondary antibody was added to well plates for 30 minutes. The substrate solution was added, followed by incubation for 30 minutes. A blocking solution was then added, and the absorbance of the samples was measured at 450 nm multi-well plate reader. The relative proliferation rates were presented as the percentage of control. BrdU assay to examine the effect of recombinant BMPs was conducted with the same manner.

### Wound healing assay and Boyden chamber assay

SMCs were gently scraped with pipette tips. They were then serum-starved for 24 hours and treated with conditioned media from macrophages that had been stimulated with BSA control, palmitate (250 µM) or they were treated with PDGF (25 ng/mL)/IL-1β (10 ng/mL). Twenty-four hours later, the wound sites along the scratches were examined and photographed at 100-fold magnification. Widths of wound gaps after 24 hours were measured on the photographs for comparison. Boyden chamber assay was conducted using 8.0-µm pore transwell inserts (Corning, Corning, NY, USA). Briefly, serum-starved SMCs were seeded in the upper chamber (250 µL, 1×10^5^ cells/well in a 24-well plate). Cell migration was stimulated by 750 µL of conditioned media or PDGF/IL-1β to the lower well of the Boyden chamber. After 4-24 hours of incubation, the surface of the upper membrane was swabbed with a cotton-tipped applicator to remove non-migrating cells. Inserts were fixed in methanol for 30 minutes and stained with 1% crystal violet for 2 hours. For quantitative analysis, the surface of membrane was eluted by methnol and optical density was measured using a microplate reader. Boyden chamber assay to test the effect of recombinant BMPs was performed with the same manner.

### Immunoblotting to assess SMC phenotype and BMP production

The effect of palmitate-stimulated macrophages on SMC phenotype was assessed by immunoblotting. Total cell lysates were collected from the cells. Cell extracts were subjected to 10% SDS-PAGE and proteins were electrotransferred to a polyvinylidene difluoride membrane for immunoblot analyses. The membrane was blocked in phosphate-buffered saline containing 0.05% Tween 20 and 5% nonfat dry milk. Immunoblotting was performed with anti-SM α-actin. The reactive bands were visualized with Supersignal West Dura Extended Duration Substrate (Thermo Scientific, Waltham, MA, USA). In the experiments using conditioned media, RAW264.7 cells were treated with BSA control or palmitate (250 µM) for 4 hours and the conditioned media were collected 20 hours later. SMCs were serum-starved for 24 hours and treated with the conditioned media or PDGF (25 ng/mL)/IL-1β (10 ng/mL) for 24 hours. Steps of immunoblotting thereafter were the same as described above. Primary antibodies were: anti-β-actin, anti-SM α-actin, and anti-SM22α. Immunoblotting to evaluate the effect of recombinant BMPs on SMC phenotypic change was performed with the same manner. BMP proteins were detected by immunoblotting in conditioned media after pull-down using heparin Sepharose beads.

### Quantitative real-time PCR

Total RNA from cells was isolated using the RNA extraction reagent Trizol (Invitrogen, Carlsbad, CA, USA). The cDNA was synthesized using Superscript III reverse transcriptase (Invitrogen) according to manufacturer's protocol. Real-time PCR was performed to determine the mRNA levels of BMP2 and BMP4 in cells using LightCycler FastStart DNA Master SYBR Green I mix (Roche Applied Science, Indianapolis, IN, USA) with a LightCycler 480 System (Roche Applied Science), in accordance with the manufacturer's instructions. The primers used were as follows: BMP2, 5′-GGTCACAGATAAGGCCATTGC-3′ (sense) and 5′-GCTTCCGCTGTTTGTGTTTG-3′ (antisense); BMP4, 5′-AGGAGGAGGAGGAAGAGCAG-3′ (sense) and 5′-GAGGAAACGAAAAGCAGAGC-3′ (antisense). GAPDH gene was used as an internal control using primers, 5′-TGGCCAAGGTCATCCATGACAAC-3′ (sense) and 5′-TCCAGAGGGGCCATCCACAGTCTTCTG-3′ (antisense). Relative quantification of each gene was calculated with the LightCycler 480 SW software (Roche Applied Science).

### Anti-BMP antibody treatment

To neutralize the effects of BMPs, we used commercially available antibodies against human BMP2 and BMP4. THP-1 cells were differentiated into macrophages using 100 nM PMA in media with 10% FBS. Three days later, the cells were washed with PBS and incubated for another 22 hours in the same medium but without FBS. On the day of the experiment, the cells were incubated for 4 hours with 250 µM palmitate or BSA control. Conditioned media were collected 20 hours later. Conditioned media were treated with 2 µg/mL of anti-BMP2, anti-BMP4 antibodies, or both for 1 hour. Antibody-treated conditioned media were added to serum-starved SMC for 24 hours.

### Transfection of siRNA

BMP2 siRNA, BMP4 siRNA and scrambled control siRNA were obtained from Santa Cruz Biotechnology (Santa Cruz, CA, USA). THP-1 cells differentiated with PMA were transfected with 10 nM of siRNA using Lipofectamine RNAiMAX Reagent (Invitrogen) according to the manufacturer's protocol. After 24 hour of siRNA transfection, we determined BMP2 and BMP4 levels in whole cell lysates by immunoblot analysis to confirm the silencing of these proteins. The cells transfected with BMP siRNA were also incubated for 4 hours with 250 µM palmitate-BSA complex or BSA control and conditioned media were collected 20 hours later.

### Statistical analysis

All data are presented as the mean ± standard error of the mean (SE). Statistical analysis between two groups was conducted using Student's *t*-test. Differences were considered statistically significant if the p value was <0.05 (2-sided). All data were analyzed using SPSS version 17.0 (SPSS Inc., Chicago, IL, USA).

## Results

### Conditioned media from palmitate-stimulated macrophages promote SMC proliferation

**Figure 1:**
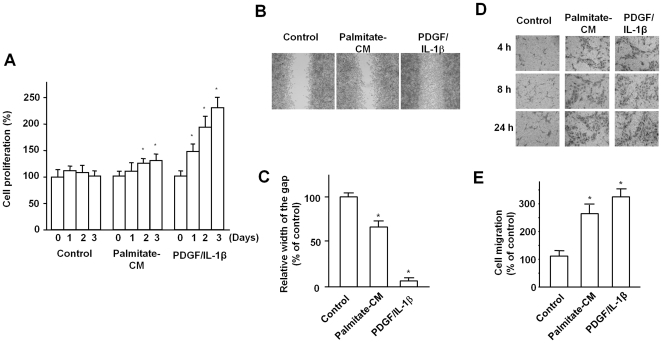
Conditioned media from palmitate-stimulated macrophages promote SMC proliferation and migration. A. To evaluate SMC proliferation, RAW 264.7 cells were stimulated with BSA control, palmitate (250 µM) for 4 hours. Conditioned media were collected 20 hours later. SMCs were serum-starved for 24 hours and treated with each conditioned media or PDGF (25 ng/mL)/IL-1β (10 ng/mL). BrdU incorporation assay showed that palmitate-conditioned media modestly increased cell proliferation compared to the control. B. In the wound healing assay, SMCs were gently scraped with a pipette tips. SMCs were serum-starved and treated with each conditioned media or PDGF/IL-1β as described in BrdU assay. Wound sites along the scratches were examined and photographed at 100-fold maginification. C. The width of the wound gap was measured on the photograph and expressed as % of control. The wound gaps were shorter in cells treated with palmitate-conditioned media or PDGF/IL-1β. D. In Boyden chamber assay, SMCs were incubated with each conditioned media or PDGF/IL-1β in the chamber for 4-24 hours. After crystal violet staining, migrated cells in the filters were observed under a microscope (Olympus, Tokyo, Japan). E. In addition, after crystal violet staining, the surface of membrane was eluted by methanol and optical density was measured using a microplate reader. Treatment with palmitate-conditioned media or PDGF/IL-1β induced significant SMC migration. Data are means ± SE of three independent experiments. *p<0.05 compared to treatment with conditioned media from BSA control.

The effects of palmitate-stimulated macrophages on SMC proliferation were evaluated by BrdU assay. Proliferation of SMCs increased 48 hours after treatment with conditioned media from RAW264.7 cells that had been stimulated with palmitate. This effect was modest compared to that of PDGF/IL-1β. The mean increase was 26–31% for palmitate and 94–132% for PDGF/IL-1β at 48–72 hours (Fig. 1A). Our results suggest that palmitate-stimulated macrophages are involved in SMC proliferation.

### Conditioned media from palmitate-stimulated macrophages promote SMC migration

The effects of palmitate-stimulated macrophages on SMC migration were assessed by a wound healing and Boyden chamber assay. In the wound healing assay, the widths of wound gaps were shorter in cells treated with palmitate-conditioned media or PDGF/IL-1β (Fig. 1B and 1C). Boyden chamber assay demonstrated that palmitate-conditioned media and PDGF/IL-1β induced significant SMC migration compared to the control (Fig. 1D and 1E). These results indicate that palmitate-stimulated macrophages are involved in promoting SMC migration.

### Conditioned media from palmitate-stimulated macrophages affect SMC phenotypes

**Figure 2:**
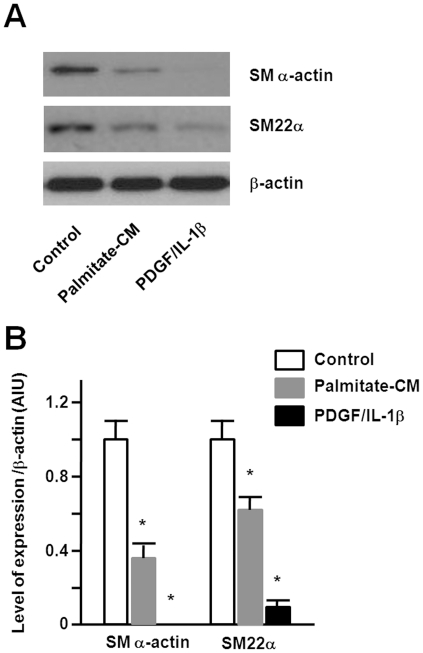
Conditioned media from palmitate-stimulated macrophages change SMC phenotype. A. RAW 264.7 cells were treated with BSA control orpalmitate for 4 hours and conditioned media were collected 20 hours later. SMCs were serum-starved for 24 hours and treated with conditioned media or PDGF/IL-1β for 24 hours. Immunoblots showed decreased SM α-actin and SM22α in cells treated with conditioned media from palmitate-stimulated macrophages. B. The band intensities were determined by quantitative densitometry. Data are illustrated in arbitrary integrator units relative to β-actin and represent the mean ± SE from three independent experiments. *p<0.05 compared to treatment with conditioned media from BSA control.

To examine the paracrine effects of palmitate-stimulated macrophages on SMC phenotype, we determined the effects of conditioned media from palmitate-stimulated macrophages on SMC contractile markers. RAW264.7 cells were stimulated with BSA control or palmitate. SMCs treated with the conditioned media from palmitate-stimulated cells or PDGF/IL-1β showed a decrease in SM α-actin and SM22α compared to those treated with the conditioned media from BSA control-treated cells (Fig. 2A and 2B).

### BMP production by palmitate-stimulated macrophages and its role in SMC proliferation, migration, and phenotypic change

**Figure 3:**
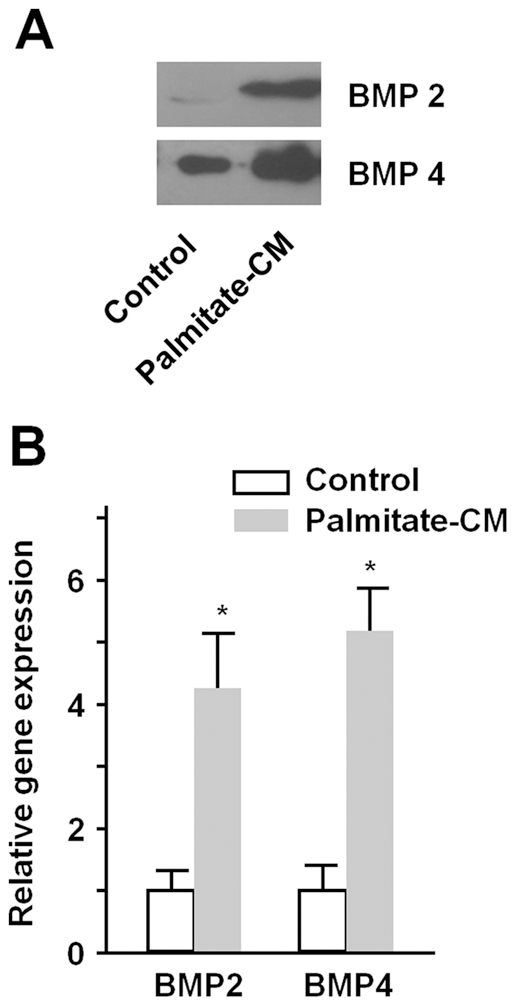
BMP production by palmitate-stimulated macrophages. A. RAW 264.7 cells were treated with BSA control or palmitate (250 µM) for 4 hours and conditioned media were collected 20 hours later. BMP 2 and 4 secreted in the media were pulled down on heparin-Sepharose columns and detected by immunoblotting. B. RAW 264.7 cells were treated with BSA control or palmitate for 4 hours, and then incubated with fresh media for 20 hours. BMP2 and BMP4 mRNA levels were analyzed by real-time PCR. Data are illustrated in arbitrary unit relative control and represent the means ± SE from three independent experiments. *p<0.05 compared to control.

**Figure 4:**
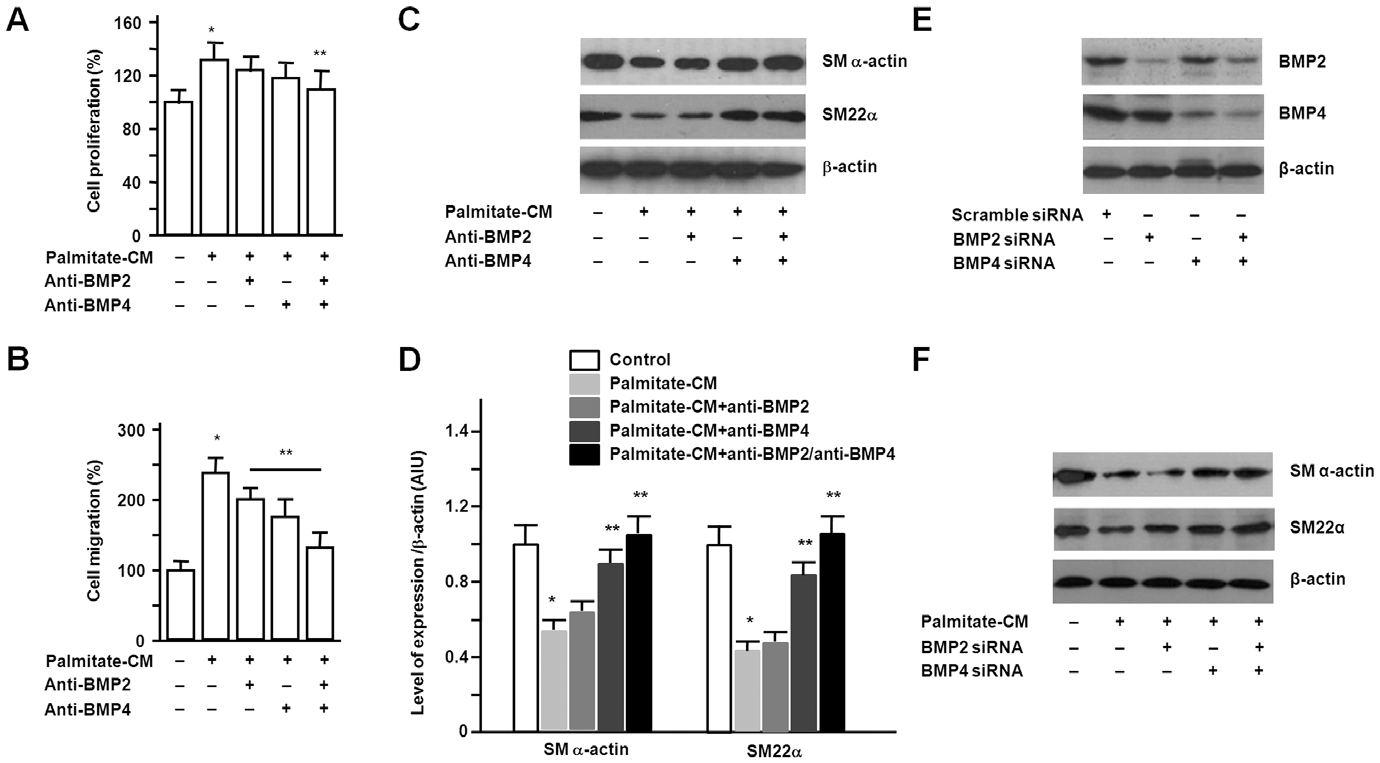
The effect of neutralization or knocking-down of BMPs on SMC proliferation, migration, and phenotypic change. A. THP-1 cells differentiated with PMA were stimulated with BSA control or palmitate for 4 hours and conditioned media were collected 20 hours later. Palmitate-conditioned media were treated without or with 2 µg/mL of anti-BMP2, anti-BMP4, or both for 1 hour. Each conditioned media was added to serum starved SMCs for 72 hours. Cell proliferation was analyzed by BrdU incorporation assay. Promotion of SMC proliferation by palmitate-conditioned media was abrogated by combined treatment of anti-BMP2/anti-BMP4. B. Each conditioned media described above was added to SMCs for 24 hours. Cell migration assay was conducted using Boyden chamber assay. Promotion of SMC migration by palmitate-conditioned media was inhibited by treatment of anti-BMP2 or anti-BMP4 or combination of both. C. Each conditioned media described above was added to SMCs for 24 hours. Immunoblots showed that anti-BMP4 or anti-BMP2/anti-BMP4 combination inhibited phenotypic change of SMCs. D. The band intensities were determined by quantitative densitometry. E. THP-1 cell-derived macrophages were transfected with BMP2 or BMP4 or both siRNA for 24 hours. Immunoblots demonstrated knockdown of BMP2 or BMP4 or both BMPs in the macrophages. F. SMCs were treated with palmitate-conditioned media from macrophages without or with knockdown of BMPs for 24 hours. Equal amount of protein were separated by SDS-PAGE, and immunoblots showed that knockdown of BMP4 or both BMPs abrogated phenotypic change of SMCs. Data are illustrated in arbitrary integrator unit relative to β-actin and represent the means ± SE from three independent experiments. *p<0.05 compared to control. **p<0.05 compared to palmitate-conditioned media treatment.

**Figure 5:**
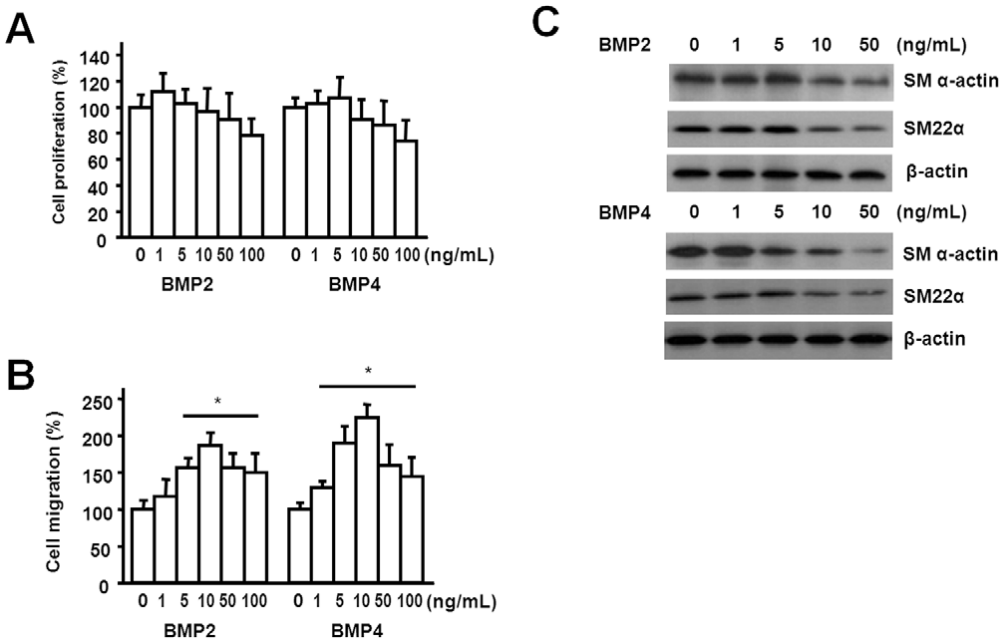
The effects of recombinant BMP2 and BMP4 on SMC proliferation, migration, and phenotypic change. A. Quiescent SMCs were treated with various concentration of BMP2 or BMP4 for 24 hours. Cell proliferation was analyzed by BrdU incorporation assay. Recombinant BMP2 and BMP4 did not have obvious effects on SMC proliferation. B. SMC migration assessment was conducted using Boyden chamber assay and BMP2 and BMP4 significantly promoted SMC migration. C. SMCs were treated with various concentration of recombinant BMP2 or BMP4 for 24 hours. Equal amount of protein was separated by SDS-PAGE and immunoblots showed both recombinant BMPs induced phenotypic change of SMCs with lowering expression of SM α-actin and SM22α.

We examined conditioned media from palmitate-stimulated macrophages to evaluate the mechanism of the effects on SMC. When stimulated with palmitate, RAW264.7 cells secreted BMP2 and BMP4 into cell culture media (Fig. 3A). In real-time PCR, mean mRNA levels of BMP2 and 4 were 4.3 and 5.2 times higher in cells treated with the palmitate-conditioned media (Fig. 3B). When palmitate-conditioned media was treated with the combination of anti-BMP2 and anti-BMP4, promotion of SMC proliferation by the media was abrogated. Anti-BMP2 or anti-BMP4 alone did not show significant effect on cell proliferation (Fig. 4A). Promotion of SMC migration by palmitate-conditioned media was inhibited by addition of anti-BMP2 or anti-BMP4 or the combination of them (Fig. 4B). To determine whether the SMC phenotypic change was BMP-dependent, we treated conditioned media from macrophages differentiated from THP-1 cells without or with addition of anti-BMP antibodies. Immunoblots showed that anti-BMP4 and anti-BMP2/anti-BMP4 antibodies inhibited the decrease of SM α-actin and SM22α (Fig. 4C and 4D). THP-1 cell-derived macrophages were transfected with BMP2 or BMP4 or both siRNA and demonstrated knockdown of BMPs (Fig. 4E). SMCs were treated with palmitate-conditioned media from macrophages without or with knockdown of BMPs. Immunoblots showed that knockdown of BMP4 or both BMPs abrogated phenotypic change of SMCs (Fig. 4F). Treatment of SMCs with recombinant BMP2 and BMP4 resulted in no apparent changes in cell proliferation (Fig. 5A). In Boyden chamber assay, however, recombinant BMP2 and BMP4 significantly promoted SMC migration (Fig. 5B). When recombinant BMP2 or BMP4 were treated to SMCs, both BMPs decreased the expression of contractile markers in a dose-dependent manner (Fig. 5C). Taken together, these results indicate that the effects of palmitate-conditioned media on SMCs are, at least in part, BMP2- and BMP4-dependent.

## Discussion

This study demonstrated that palmitate influences the paracrine effects of macrophages. Palmitate-stimulated macrophages promoted SMC proliferation and migration. In addition, these macrophages decreased the levels of SMC contractile markers, indicating that SMCs were changed into more synthetic forms. These effects of palmitate-stimulated macrophages required the secretion of BMP2 and BMP4, as demonstrated by the attenuation of SMC proliferation, migration, and phenotypic changes after treatment with neutralizing antibodies against BMPs or knockdown of BMPs with siRNA. The influences of these proteins were further confirmed by direct treatment of recombinant BMP2 and BMP4 on SMCs. The effect of BMP4 was more consistent compared to that of BMP2. Particularly, the effects of BMPs on SMC migration or phenotypic change were more obvious, whereas their effect on SMC proliferation seemed not significant or modest at maximum. Collectively, these findings suggest a new mechanism linking elevated saturated fatty acids and the progression of vascular diseases, possibly mediated by BMP2 and BMP4 originating from macrophages.

Prior studies have provided evidence for a proinflammatory effect of saturated fatty acids, particularly palmitate. In mouse and human cells, palmitate induces cyclooxygenase-2 [2], IL-6 [3], [15], TNF-α [4], IL-8 [4], [15], and IL-1β [4]. Here, we focused on BMPs and found that BMP2 and BMP4 were produced at higher levels by palmitate-stimulated macrophages compared to control macrophages. Our results are in agreement with those of a recent study showing that the incubation of human endothelial cells with palmitate increases BMP4 production, and that 6 weeks of a high fat diet elevated mRNA levels of BMP4 in mouse thoracic aorta [16]. Toll-like receptor 4 is known to link fatty acids and inflammation [17] and toll-like receptor 4^−/−^ mice fed a high fat diet do not induce vascular BMP4 [16]. However, the mechanism of BMP production by palmitate-stimulated macrophages is unclear, and is beyond the scope of our current study. To our knowledge, the present study is the first to show that palmitate-stimulated macrophages produce BMPs possessing important paracrine effects. Furthermore, we observed similar effects of conditioned media from stimulated RAW264.7 cells and THP-1 cell-derived macrophages. Based on these findings, the effect of palmitate might not be limited to cells from a single species.

We found that BMP4 played a role in changing SMC phenotype to the synthetic form. Several groups of investigators have documented the effect of BMP4 in vascular cells. Sorescu et al. reported that BMP4 produced in endothelial cells stimulates monocyte adhesion which is dependent on NFκB and intercellular adhesion molecule 1 [9]. The effects of BMP4 on SMC have been examined by previous studies, mainly in the pulmonary vasculature, but these findings have been inconsistent and context and/or site-dependent. In a study of neointimal hyperplasia, endothelial BMP4 expression was upregulated after carotid ligation in mice and activated BMP4 signaling resulted in decreased SMC proliferation and migration [18]. BMP4 inhibited proliferation of SMCs isolated from proximal pulmonary arteries, while it stimulated that of SMCs from peripheral arteries [10]. In another study, pulmonary microvascular endothelial cells secreted BMP4 in response to hypoxia and promoted proliferation and migration of vascular SMCs [11]. In addition, two recent studies reported that BMP2, 4, and 6 can regulate vascular SMC phenotypic change through myocardin-related transcription factors [19], [20].

BMP2 expression was found to be upregulated in atherosclerotic lesions [7]. Nakaoka et al. showed that overexpression of BMP2 using adenovirus results in reduced neointimal proliferation in a rat carotid injury model [21]. However, Anderson et al. found that BMP4-deficient mice have reduced vascular SMC proliferation and vascular remodeling, while there is no change in vascular SMCs or vessels in BMP2-deficient mice under hypoxic conditions [12]. In our study, BMP2 production increased in macrophages stimulated with palmitate. We found that BMP2 also had significant influence on the migration and phenotypic change of SMCs, although the effect was less consistent than that of BMP4.

We note that our study has some limitations. First, a variety of inflammatory mediators including growth factors, interleukins, and TNF-α are involved in SMC phenotypic change, growth, and migration [5], [22]. Therefore, we cannot rule out the possibility that other cytokines released from palmitate-stimulated macrophages could also have affected SMCs. However, because we demonstrated the effect of BMPs using neutralizing antibody, gene knockdown, and direct treatment of recombinant proteins, it is clear that BMP2 and 4 play a significant role in SMCs. Second, we evaluated the effects of conditioned media from palmitate-stimulated RAW264.7 cells and THP-1 cell-derived macrophages. SMC responses to stimulants can depend on cell types, origins of cells, and culture conditions. In this regard, although we evaluated the effect of conditioned media from two kinds of macrophages, further studies are required to extend these data to other biological settings and translate them to human vascular pathophysiology.

In conclusion, palmitate-stimulated macrophages promoted vascular SMC proliferation and migration, and changed SMCs to a more synthetic form. The effects of these stimulated macrophages on SMC migration and phenotypic change were mediated, at least in part, by BMP2 and BMP4. Our results suggest a novel mechanism linking elevated saturated fatty acid levels and the progression of vascular diseases, possibly mediated by BMPs produced by macrophages.
